# Papillary muscle infarction and cardiovascular outcomes

**DOI:** 10.1186/1532-429X-13-S1-O89

**Published:** 2011-02-02

**Authors:** Geetha P Bhumireddy, Nikolas Krishna, Nripen Donneti, On Chen, Ijaz Ahmad, Quaratal Jamell, Joshua Socolow, Sorin J Brener, Igor Klem, Joshua Fogel, Terrence Sacchi, John F Heitner

**Affiliations:** 1 New York Methodist Hospital, Brooklyn, NY, USA; 2 New York Methodist Hospital, New York, NY, USA; 3 Duke Medical Center, Durham, NC, USA

## Introduction

Recent studies suggest that papillary muscle infarction (PMI) detected by contrast enhancement cardiac magnetic resonance (ce-CMR) may correlate with mitral regurgitation and reduced left ventricular ejection fraction (LVEF). However, there is scant data about the association of PMI with cardiovascular outcomes.

## Purpose

To determine the prognostic significance of PMI, detected by ce-CMR, in patients with ischemic (ICM) and non-ischemic cardiomyopathy (NICM).

## Methods

We evaluated the images of 456 consecutive patients who underwent ce-CMR for the assessment of left ventricular function and viability. PMI (both antero-lateral and postero-medial papillary muscles), ICM and NICM were assessed by visual assessment by interpreters blinded to clinical data. All patients had a LVEF ≤50%. ICM and NICM were determined by presence and pattern of hyper-enhancement. Patients were followed by telephone interview for the following outcomes: all-cause mortality, exacerbation of CHF, defined as a decrease in NYHA functional class, myocardial infarction or re-hospitalization due to a cardiac etiology.

## Results

Our study population consisted of 320 patients with ICM and 136 with NICM (mean age: 63 years; males: 66%). The prevalence of PMI was 66% and 12% in the ICM and NICM patients, respectively. There was a higher prevalence of postero-medial PMI compared to antero-lateral PMI in both the ICM and NICM (66% vs. 30% and 63% vs. 38% respectively). Mean follow up was 2.8 ± 1.8 years.

Presence of PMI was associated with a significantly higher incidence of all-cause mortality and cardiovascular outcomes (11% vs. 4%, p=0.02 and 54% vs. 42% p=0.009, respectively). Patients with both papillary muscles infarcted had a higher incidence of all-cause mortality and cardiovascular outcomes compared to patients with no PMI (30% vs. 4%, p<0.01; 53% vs. 42%, p<0.05). In addition, postero-medial PMI was associated with a higher incidence of cardiovascular outcomes and worsening of CHF compared to patients with no PMI (57% vs. 42%, p<0.05; 38% vs. 27%, p=0.019). After multiple logistic regression analysis, both PMI and poster-medial PMI were independent predictors of cardiovascular outcomes, and patients who had both papillary muscles infarcted had a higher incidence of death (Table 1).

**Table 1:** Univariate and multivariate logistic regression anlyses of cardiovascular (CV) outcomes

Variable	Univariate analysis (Odds Ratio)	95% CI	P-value	Multivarites analysis (Odds Ratio)	95% CI	P-value
**Papillary muscle infarction**						

Death	3.39	1.49-7.67	0.003	2.05	0.82-5.0	0.12
Combined CV outcome	1.64	1.13-2.37	0.09	1.53	1.01-2.32	0.04

**Stratified by location and number of papillary muscle infarctions**						

**Both postero-medial and antero-lateral**						

Death	11.66	4.9-29.9	<0.001	7.09	2.35-29.35	<0.001
Worsening CHF	0.63	0.29-1.33	0.25	0.50	0.21-1.17	0.11
Combined CV outcome	1.56	0.83-2.93	0.16	1.32	0.66-2.63	0.43

**Postero-medial**						

Death	1.6	0.96-2.64	0.68	1.84	0.9-2.8	0.7
Worsening CHF	1.65	1.04-2.61	0.03	1.60	0.96-2.64	0.06
Combined CV outcome	1.84	1.19-2.84	0.006	1.73	1.07-2.77	0.02

**Antero-lateral**						

Death	3.06	0.95-9.77	0.06	2.16	0.61-7.62	0.23
Worsening CHF	0.67	0.31-1.42	0.29	0.63	0.28-1.38	0.25
Combined CV outcome	1.30	0.68-2.34	0.44	1.25	0.65-2.39	0.49

**Clinical variables**				**Multivariate Analysis for Death**		

Age				1.031	0.99-1.06	0.008
Gender				1.47	0.63-3.41	0.37
CHF				1.59	0.66-3.76	0.29
Myocardial infarction				1.30	0.56-3.0	0.54
Valve Dysfunction				2.54	1.1-5.39	0.029
Ejection Fraction				0.97	0.94-1.0	0.05
Smoking				0.43	0.05-3.38	0.42
Hypertension				0.87	0.33-2.25	0.77
Diabetes				1.43	0.65-3.12	0.36
Revascularization				0.88	0.37-2.08	0.77

## Conclusions

Papillary muscle infarction and postero-medial PMI are independent predictors of combined cardiovascular outcomes. Postero-medial PMI is more prevalent than antero-lateral PMI and is also an independent predictor of worsening CHF.

